# Supplementary material for: Multi‐dataset identification of innovative feature genes and molecular mechanisms in keratoconus
Source: J Cell Mol Med. 2024 Sep 19;28(18):e70079. doi: 10.1111/jcmm.70079 (PMC11412914; doi:10.1111/jcmm.70079)
Supplement: Supplementary file 2 — Material S2. [file JCMM-28-e70079-s002.zip › 7.GSEA analysis/6.SNORA74B/KEGG_BASAL_CELL_CARCINOMA.html]

Details for gene set KEGG\_BASAL\_CELL\_CARCINOMA[GSEA]

|  || Dataset | SNORA74B.group.cls#h\_versus\_l |
| Phenotype | group.cls#h\_versus\_l |
| Upregulated in class | l |
| GeneSet | KEGG\_BASAL\_CELL\_CARCINOMA |
| Enrichment Score (ES) | -0.59345096 |
| Normalized Enrichment Score (NES) | -1.7412606 |
| Nominal p-value | 0.0 |
| FDR q-value | 0.5927988 |
| FWER p-Value | 0.286 |
Table: GSEA Results Summary

  

Fig 1: Enrichment plot: KEGG\_BASAL\_CELL\_CARCINOMA      
 Profile of the Running ES Score & Positions of GeneSet Members on the Rank Ordered List

  

| SYMBOL | TITLE | RANK IN GENE LIST | RANK METRIC SCORE | RUNNING ES | CORE ENRICHMENT || 1 | PTCH2 | na | 7369 | 0.152 | -0.1961 | No |
| 2 | WNT16 | na | 8300 | 0.129 | -0.2090 | No |
| 3 | WNT10B | na | 8944 | 0.115 | -0.2151 | No |
| 4 | PTCH1 | na | 9661 | 0.099 | -0.2250 | No |
| 5 | WNT5A | na | 10441 | 0.084 | -0.2384 | No |
| 6 | WNT8A | na | 12290 | 0.056 | -0.2856 | No |
| 7 | WNT3 | na | 12537 | 0.053 | -0.2870 | No |
| 8 | TCF7L2 | na | 12663 | 0.051 | -0.2851 | No |
| 9 | WNT2 | na | 14134 | 0.034 | -0.3238 | No |
| 10 | SUFU | na | 14239 | 0.033 | -0.3232 | No |
| 11 | FZD1 | na | 14343 | 0.032 | -0.3227 | No |
| 12 | HHIP | na | 14705 | 0.028 | -0.3302 | No |
| 13 | FZD5 | na | 15466 | 0.020 | -0.3499 | No |
| 14 | FZD6 | na | 16365 | 0.011 | -0.3747 | No |
| 15 | CTNNB1 | na | 17783 | -0.003 | -0.4152 | No |
| 16 | GSK3B | na | 17871 | -0.004 | -0.4172 | No |
| 17 | FZD9 | na | 18101 | -0.007 | -0.4230 | No |
| 18 | WNT4 | na | 18195 | -0.008 | -0.4248 | No |
| 19 | WNT3A | na | 18601 | -0.013 | -0.4351 | No |
| 20 | FZD3 | na | 18707 | -0.014 | -0.4366 | No |
| 21 | TP53 | na | 19572 | -0.024 | -0.4590 | No |
| 22 | WNT9A | na | 20087 | -0.029 | -0.4707 | No |
| 23 | GLI3 | na | 20392 | -0.033 | -0.4759 | No |
| 24 | AXIN1 | na | 20829 | -0.038 | -0.4844 | No |
| 25 | DVL3 | na | 20946 | -0.039 | -0.4835 | No |
| 26 | DVL1 | na | 21883 | -0.050 | -0.5050 | No |
| 27 | APC2 | na | 22646 | -0.060 | -0.5205 | No |
| 28 | FZD4 | na | 23557 | -0.074 | -0.5388 | No |
| 29 | STK36 | na | 24838 | -0.096 | -0.5654 | No |
| 30 | WNT2B | na | 25314 | -0.104 | -0.5679 | No |
| 31 | FZD8 | na | 25688 | -0.111 | -0.5667 | No |
| 32 | DVL2 | na | 26618 | -0.130 | -0.5794 | Yes |
| 33 | WNT10A | na | 26979 | -0.139 | -0.5747 | Yes |
| 34 | TCF7L1 | na | 27185 | -0.144 | -0.5651 | Yes |
| 35 | SMO | na | 27678 | -0.156 | -0.5624 | Yes |
| 36 | GLI2 | na | 27694 | -0.157 | -0.5460 | Yes |
| 37 | FZD10 | na | 27856 | -0.161 | -0.5332 | Yes |
| 38 | TCF7 | na | 28810 | -0.189 | -0.5403 | Yes |
| 39 | WNT7B | na | 28850 | -0.190 | -0.5208 | Yes |
| 40 | WNT8B | na | 29836 | -0.219 | -0.5255 | Yes |
| 41 | APC | na | 30020 | -0.226 | -0.5064 | Yes |
| 42 | WNT9B | na | 30647 | -0.250 | -0.4974 | Yes |
| 43 | WNT7A | na | 31601 | -0.301 | -0.4924 | Yes |
| 44 | WNT1 | na | 32208 | -0.335 | -0.4737 | Yes |
| 45 | WNT6 | na | 32236 | -0.336 | -0.4381 | Yes |
| 46 | FZD2 | na | 32364 | -0.345 | -0.4045 | Yes |
| 47 | WNT5B | na | 32480 | -0.353 | -0.3697 | Yes |
| 48 | GLI1 | na | 32735 | -0.371 | -0.3370 | Yes |
| 49 | BMP4 | na | 33250 | -0.419 | -0.3066 | Yes |
| 50 | FZD7 | na | 33265 | -0.421 | -0.2615 | Yes |
| 51 | BMP2 | na | 33393 | -0.437 | -0.2180 | Yes |
| 52 | SHH | na | 33951 | -0.524 | -0.1775 | Yes |
| 53 | AXIN2 | na | 34084 | -0.552 | -0.1217 | Yes |
| 54 | LEF1 | na | 34351 | -0.633 | -0.0609 | Yes |
| 55 | WNT11 | na | 34430 | -0.665 | 0.0086 | Yes |
Table: GSEA details [plain text format]

  

Fig 2: KEGG\_BASAL\_CELL\_CARCINOMA      
 Blue-Pink O' Gram in the Space of the Analyzed GeneSet

  

Fig 3: KEGG\_BASAL\_CELL\_CARCINOMA: Random ES distribution      
 Gene set null distribution of ES for **KEGG\_BASAL\_CELL\_CARCINOMA**
